# Internet of Things (IoT)-enabled framework for a sustainable Vaccine cold chain management system

**DOI:** 10.1016/j.heliyon.2024.e28910

**Published:** 2024-03-28

**Authors:** Shaojun Jiang, Sumei Jia, Hongjun Guo

**Affiliations:** Hebei Key Laboratory of Optical Fiber Biosensing and Communication Devices (SZX2022010), Institute of Information Technology, Handan University, Handan, 056005, China

**Keywords:** Vaccine cold chain, Vaccine cold chain management, Temperature monitoring, Internet of Things, Vaccine cold chain equipment

## Abstract

Vaccines are a unique category of drugs sensitive to temperature and humidity and whose effectiveness directly impacts public health. There has been an increase in vaccine-related adverse events worldwide, particularly in developing countries, attributed to suboptimal temperatures during transport and storage. At the same time, the Internet of Things (IoT) has ushered in a paradigm shift in vaccine information and storage monitoring, enabling continuous 24/7 tracking. This further reduces the dependence on limited human resources and significantly reduces the associated errors and losses. This paper presents an IoT-driven framework that aims to improve the sustainability of medical cold chain management. The framework promotes trust and transparency in vaccine surveillance data by accessing and authenticating IoT devices. The proposed system aims to improve the safety and sustainability of vaccine management. Moreover, we provide detailed insights into the design and hardware components of the proposed framework. In addition, the specific use of the framework in a particular province is highlighted, covering the design of the software platform and the analysis of the hardware equipment.

## 1 Introduction

Vaccines and a certain category of drugs are among the most cost-effective tools for preventing infectious diseases [1,2]. The World Health Organization (WHO) found vaccinations can prevent two to three million deaths yearly [3]. Vaccination is estimated to have reduced mortality rates for nine diseases by an average of 97.8% [4]. The ongoing COVID-19 outbreak over the past three years has profoundly impacted our lives and the global landscape [5]. This virus has claimed millions of lives worldwide [6]. Vaccination is a crucial solution to combat the pandemic [7,8]. Nevertheless, it is crucial to strictly control the temperature of vaccines from production to administration by healthcare professionals [9]. At extreme temperatures, whether too hot or too cold, vaccines can quickly lose their effectiveness, especially during transport and storage [10]. Currently, the recommended storage conditions for vaccines in China are a temperature range of 2–8 °C. This ensures that the vaccine retains maximum effectiveness from production through the cold chain to storage and vaccination. The cold chain is a term used to refer to the supply chain in which goods, such as vaccines, are maintained in a temperature-controlled environment [11].

Therefore, the question arises: Is storing vaccines in freezers safe? To find the answer, we describe various incidents related to vaccine refrigeration and examine global cases where COVID-19 vaccines were not stored properly. There are numerous reports of vaccine wastage due to temperature fluctuations. For example, on January 20, 2021, Michigan authorities announced that nearly 12,000 doses of Moderna's COVID-19 vaccine were at risk due to a failure in temperature control during transportation [12]. In Maine, over 16,000 doses were wasted, while in Milwaukee, an employee intentionally removed and destroyed over 500 doses [13]. In January 2021, approximately 2000 doses of the Moderna vaccine were damaged at a Boston hospital. Additionally, ABC News attributed this to a cleaner turning off a refrigerator containing the vaccines. According to the Centers for Disease Control and Prevention, the Moderna vaccine can be stored at 36–46 °F (2–8 °C) for up to 30 days [14]. That same month, over 1100 Pfizer vaccine doses were lost in Florida when a healthcare worker accidently closed a specialized refrigerator. The Pfizer vaccine has strict storage requirements: 21 °C (−70 °F). If the vaccine is left at room temperature or at higher temperatures for more than five days, it will degrade [15]. In April 2021, a cleaner in Kyrgyzstan unplugged a refrigeration unit to charge her phone, throwing nearly 1,000 COVID-19 vaccine doses away [16]. Additionally, a mishap in Yokohama, Japan, in May 2021 resulted in the accidental administration of 119 improperly stored doses after 366 doses were removed from a freezer but not cooled adequately due to a disconnected refrigerator. The remaining 247 doses were discarded. Notably, Japan threw away at least 7000 doses due to improper storage [17]. These events have raised international concerns about the timely and safe administration of vaccines. Despite the notable importance of vaccines cold chain, end-users have no access to information, including vaccine conditions and temperature along the vaccine supply chain. There is often no transparency and routine monitoring in the cold chain system. Moreover, due to the lack of accountability, vaccines may get lost or stolen in any part of the supply chain. These issues contribute to losing about 30% of vaccines during transportation. In recent years, with the rapid development of IoT technology, it has become a necessary technical means of vaccine cold chain management. By combining IoT technology with vaccine cold chain management, real-time monitoring, traceability and management of the vaccine transportation process can be achieved to ensure the safety and effectiveness of vaccines.

### 1.1 Motivation

From the above incidents, it is clear that the main reason for vaccine wastage is due to inadequate storage and transportation practices [18,19]. There is a gap in real-time monitoring of the environments where vaccines are stored. When these conditions change, vaccination managers are often not notified in a timely manner, resulting in significant vaccine damage.

The World Health Organization (WHO) has offered guidelines concerning vaccine “cold chain” management [20,21]. The term "cold chain" denotes a supply chain where products like vaccines are consistently maintained in a temperature-controlled setting [22,23]. This process ensures vaccines' temperatures are monitored from production to administration at healthcare facilities. However, despite the critical nature of maintaining the vaccine cold chain, end-users remain uninformed about specific conditions, including temperatures, along the vaccine's journey. There is typically a need for more transparency and regular oversight within cold chain systems. This deficiency, coupled with potential theft or misplacement due to inadequate accountability, results in an estimated 30% of vaccines being lost during transit [24].

### 1.2 Contribution

The main contribution of this paper is to provide a comprehensive understanding of the issues on the vaccine cold chain since the COVID-19 outbreak, and this study elucidates the following research questions (RQs).

RQ 1 What are the key issues of vaccine supply analysed through the novel coronavirus pneumonia epidemic?

RQ 2 How can current technologies and methods be applied to solve these problems?

RQ 3 How to build a system framework to better meet the needs of vaccine cold chain management?

RQ 4 How to develop relevant hardware and software products to meet the implementation of a system framework?

RQ 5 How to implement and evaluate the effectiveness of the system framework?

**Fig. 1:**
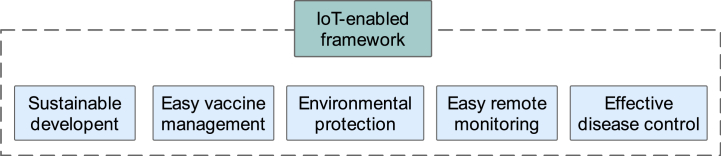
IoT-enabled framework advantages.

By analysing practical case applications, this paper provides the Internet of Things (IoT)-enabled framework for sustainable vaccine cold chain management that leverages IoT's benefits. This technology enables remote monitoring and management of all cold storage units, freezers, refrigerators, and delivery incubators in storage areas of hospitals, pharmacies, and medical wards. All collected data is integrated into the system management software via medical IoT, enabling real-time monitoring, display, and recording of temperature and humidity metrics. This system also makes it easier to query historical data. If irregularities occur, the system immediately notifies vaccine administrators so they can resolve the issue in real-time. This prevents the breakdown of valuable medication and reduces possible significant losses. All vaccination-related data is stored in cloud databases to meet various needs, such as the review of vaccination records by individuals, the assessment of vaccination progress by management, and the assessment of vaccine effectiveness and waste by regulators. Fig. 1 shows the main benefits of this IoT-driven framework.

### 1.3 Organization

The rest of the paper is organized as follows: Section 2 provides the literature review. Section 3 provides the background for the study. Section 4 discusses related work in this area. Section 5 presents the IoT temperature monitoring devices. Section 6 describes the framework of the sustainable vaccine cold chain management system. Section 7 presents the proof of concept for IoT-enabled framework. Section 8 provides a detailed analysis of the proposed IoT-based framework. Finally, we conclude the study in Section 9.

## 2 Literature review

Due to the significant role of vaccines in preventing the outbreaks of infectious diseases, especially since the outbreak of COVID-19 in 2019, it has had a significant impact on people's production and life, and vaccines have played a crucial role in solving practical problems. In order to improve the effectiveness and safety of vaccines, more and more researchers have recently become interested in and studied the vaccine cold chain from various perspectives. Table 1 presents some research on vaccine cold chain proposed in the literature.

**Table 1:** List of literature.

Publications	Research content	Transportation	Monitoring	Management	Distribution
Qi Lin et al [25]. 2020	The authors analysed the importance of vaccine cold chain transportation decision-making, and established corresponding models to analyse events and improve vaccine safety.	✓		✓	
Thakur et al. [26] 2024	The authors study an effort to explore the factors of vaccine cold chain management to develop a sustainable and resilient healthcare delivery system in any health outbreak.			✓	
Nugroho et al. [27] 2021	This paper discusses how vaccine cold chain management and cold storage technology can address the challenges of vaccination programs.		✓	✓	
Ronan et al. [18] 2021	The authors describe current IoT temperature monitoring devices and propose Blockcoldchain to track vaccine cold chains using blockchain.		✓		✓
Vijaya et al. [28] 2021	The author expands on existing models and devises an approach that considers the needed extensive distribution capabilities and special storage requirements of vaccines while being cognizant of costs.they provide decision support on distributing the vaccine to an entire population based on priority.				✓
Verónica et al. [19] 2020	A vaccine cold chain temperature monitoring study was conducted using standard WHO study protocol to document potential problems and identify appropriate control measures. Multiple temperature monitoring devices were used in the study to evaluate user-friendliness of these devices and staff attitudes towards them.		✓		
Erassa et al. [29] 2023	The paper aimed to assess vaccine cold chain management and associated factors at public health facilities and district health offices.			✓	
Umit et al. [30] 2022	The authors review the risks associated with the vaccine cold chain that require temperature monitoring throughout shipment and storage. Electronic and chemical monitoring devices are compared along with data needs. Regulatory oversight and guidance are also discussed.		✓	✓	
Shanmukhi et al. [31] 2023	The authors present a decision support framework for optimizing multiple aspects of vaccine distribution across a multitier cold chain network. Vaccine transportation and administration lead times are also incorporated within the models.	✓		✓	✓
Eyüp et al. [32] 2023	This paper investigates the distribution problem of the COVID-19 vaccine at the provincial level in Turkey and the management of medical waste, considering the cold chain requirements and the perishable nature of vaccines. It presents a novel multi-period, multi-objective, mixed-integer linear programming model for solving the distribution problem.				✓
Di et al. [33] 2022	This paper studies the risk assessment of vaccine cold chain under the background of significant epidemics, takes vaccine cold chain logistics as the research object, analyzes the risk factors of each link, explores the opportunities and challenges in the Internet of Things environment, and then realizes the efficient development of vaccine cold chain logistics.		✓	✓	
Xu et al. [34] 2022	The authors develop a temperature monitoring system by using narrow-band Internet of Things (NB-IoT) technology, and the control unit sends the data collected by sensors to the cloud platform of the Internet of Things to realize the real-time temperature monitoring during the whole process of vaccine cold chain transportation.	✓	✓	✓	
Zuo et al. [35] 2022	This paper studies the problem of the performance of UHF RFID tags in the dense environment of vaccine transportation; the research results have certain guiding significance for the label design and the application of the label in the cold chain transportation of vaccines.	✓	✓	✓	
Wang et al. [36] 2022	This article is based on edge-cloud synergy technology, studied a new type of intelligent vaccine cold chain system architecture, the Internet of thing send-side-cloud architecture, cloud center reserves vast amounts of vaccine information, can do big data analysis, the edge gateway deployment in the vaccine cold chain inside the car, built-in edge gateway servers and databases, keep standard vaccine information and preservation conditions.		✓	✓	
Amit et al. [37] 2022	The authors examine the potential barriers to blockchain for COVID-19 vaccine management and analyse why blockchain technology has not been fully adopted for vaccine distribution and management. This study shows that the requirement of change in organizational structure and policies is the most prominent barrier, and the barrier related to the requirement of large-scale IoT infrastructure and lack of technical expertise are the most impactful barriers.		✓	✓	✓
Hui Hu et al. [38] 2023	This study establishes an intelligent VSC management system that provides decision support for VSC management during the COVID-19 pandemic. The system combines blockchain, the internet of things (IoT), and machine learning that effectively address the three issues: vaccine quality, demand forecasting, and trust among stakeholders—persist in the vaccine supply chain(VSC).		✓	✓	
Shashank et al. [39] 2022	The analysis illustrates the positive influence of IoT adoption on the performance of the VSC in distributing the COVID vaccine. The finding also shows the positive relationship between product, supply, demand, and social behavior in IoT adoption. Analysis displays that Indian politicians can substantially impact vaccine distribution because they have influence and awareness of their local districts.		✓	✓	✓

As shown in Table 1, all the studies cover the transportation, distribution, management, and monitoring of the vaccine cold chain, and each study analyses the problems from one or several aspects and proposes corresponding solutions to improve the science and safety of the vaccine cold chain, as well as the effectiveness of vaccines. However, the vaccine cold chain is a system engineering, and very few kinds of literature study the framework of the vaccine cold chain. Even if the intelligent vaccine cold chain system architecture based on edge-cloud collaboration technology is mentioned in Literature 33, the literature only proposes the composition and role of the overall framework. However, it does not propose the specific implementation of the system, and there is no practical application, so there is no analysis of the actual effect. This paper analyses the main problems of the vaccine cold chain from the whole life cycle of vaccines, and proposes an Internet of Things (IoT)-enabled framework for a sustainable vaccine cold chain management system from practical application cases, introduces the overall structure of the system framework, details the specific design scheme of each part and the corresponding technology used, which also contains the hardware and software design of data-supporting IoT terminal devices, and this paper analyses the actual cases of the specific implementation program design and feasibility.

However, the research of this thesis is still insufficient. The application of blockchain technology in vaccine cold chain management has been studied in literature 26, 37, 38, and the application model of blockchain technology in vaccine cold chain management has been studied in literature 26; the application of blockchain and machine learning combined with IoT in vaccine cold chain management has also been studied in literature 38; even though literature 37 has studied the obstacles to the application of regional block technology in vaccine cold chain and studied and analysed the detailed barriers and gave the corresponding solution ideas. In summary, it can be seen that blockchain, despite its barriers to its use, will play a great role in enhancing vaccine cold chain management in the future. However, in this thesis, the research mainly proposes a vaccine cold chain management framework, designs specific design solutions for hardware equipment and software equipment that support the implementation of the framework, and also studies the specific implementation of the framework. However, the analysis of the specific impact on vaccine cold chain management after the use of the framework is insufficient, and there is no introduction of blockchain technology, machine learning and other technologies proposed in the above literature to analyse and learn from the data generated in the use of the framework to provide feedback for better optimization of vaccine cold chain management.

## 3 Background

### 3.1 Cold chain

The WHO states, "Vaccination is a simple, safe, and effective method to protect individuals from harmful diseases before they encounter them" [28]. However, these vaccines are very sensitive to temperature fluctuations and must be kept within certain temperature parameters. For example, the WHO has set a temperature guideline of +2 to +8 °C for storage and transport for certain vaccines. Deviation from this range, even for a short time, can cause vaccines to become ineffective. Some vaccines have even stricter requirements, requiring temperatures as low as −80 °C, requiring specialized monitoring solutions.

**Fig. 2:**
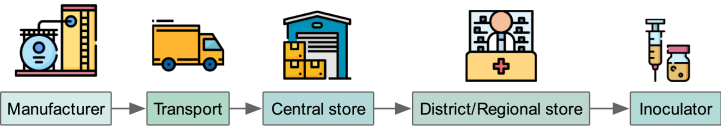
*Cold chain* path.

The term "cold chain" refers to a continuous, temperature-regulated supply chain ensuring vaccines are maintained in their required environment. Fig. 2 illustrates the cold chain process as defined by WHO. At the abstract level, the journey begins with vaccine production by the manufacturer. These vaccines are then transported to an airport or another transportation hub and sent to a central storage facility. From there, these vaccines are distributed to regional storage units and, finally, to health centers, where they are administered to individuals [19].

This research focuses on IoT systems to monitor these cold chains. Several units, such as data loggers, temperature-humidity sensors, and low-energy transmission devices, are integral to this monitoring process. It is important to note that the equipment/unit used in cold chains can vary.

At the national level, the cold chain can consist of cold rooms, freezers, standard freezers, refrigerators, and refrigerated boxes, depending on the quantity of vaccines required. In certain cases, refrigerated vehicles can also be used for transport. At a more local, intermediate level - such as a district or region - cold chains typically include cold and freezer rooms, chest freezers, refrigerators, and refrigerated boxes, with refrigerated vehicles sometimes serving as transport. Finally, the most important units for maintaining the cold chain in health centers are refrigerators, often accompanied by freezer or cooler compartments for water bags, coolers, and portable vaccine containers.

### 3.2 IoT technology

IoT has become attractive for real-time tracking, product management, security, and continuous monitoring [40,41]. Different IoT technologies are being actively trialed in a wide range of supply chain applications including cold chains, perishable products, agriculture and crops, and some manufacturing supply chains [42]. Advanced IoT devices—smartphones, wearables, and near-field communication tools—with sensors and actuators significantly improve intelligent communications, monitoring, event tracking, and technical analysis [43]. Numerous studies have highlighted the potential of IoT devices to improve vaccine cold chain management by providing better monitoring, increased transparency, reliability, and adaptability, all of which are critical for effective vaccine distribution and monitoring. The use of IoT for intelligent medical cold chain monitoring can facilitate remote monitoring and management of cold storage, freezers, refrigerators, and insulated shipping containers distributed across storage rooms in hospitals, pharmacies, and medical facilities [44]. All monitored data are integrated into the system management software via medical IoT, ensuring real-time observation, display, and recording of temperature and humidity metrics. Moreover, these IoT-based systems provide access to historical data and help avoid significant losses due to degradation or mishandling of expensive medicines and other vital substances.

## 4 Related work

In this section, we focus on the application of IoT technology in supply chains and vaccine traceability. As the benefits of IoT become evident in various sectors, its adoption is increasing, especially in areas that require secure handling and processing of insecure information [45]. In fact, IoT can facilitate data storage and sharing within the vaccine distribution network, thereby simplifying authenticity. In addition, IoT ensures data integrity [46], maintains trust between stakeholders, and simultaneously ensures end-to-end traceability [47].

The use of IoT technology in the vaccine cold chain enables comprehensive monitoring of the entire journey of the vaccine. This includes monitoring the temperature and humidity in the vaccine production environment, information about transport equipment, data on temperature and moisture during transport, and conditions in central and regional vaccine storage facilities. In addition, the technology can monitor device data and the temperature and humidity in recent vaccination centers. This system can seamlessly merge with the vaccination centers' data platforms and gives vaccinators access to all relevant vaccine information. At the same time, health authorities can better identify the causes of any vaccine side effects. The countless benefits of IoT technology improve supply chain efficiency and reliability and address challenges related to trust within the supply chain. This research highlights that among the influencing factors highlighted, traceability is the most critical factor in ensuring world-class vaccine management. In summary, leveraging the multiple benefits of IoT technology is of utmost importance.

Unlike other studies, our solution provides a detailed introduction to system architecture design, software and hardware design, and system implementation. Based on use cases, we also highlight how IoT technology can provide technical support for the sustainability of cold chain management.

## 5 IoT devices and overall system software

The ambient temperature primarily influences the integrity of vaccines within the cold chain. Maintaining an optimal temperature is crucial to prolong the effectiveness of vaccines and ensure their quality. Therefore, continuous monitoring of temperature data during vaccine transportation and storage is essential. This requires the development and deployment of IoT-centric hardware and software and the establishment of an information system. This system should provide data collection and accessibility from vaccine production to administration, coupled with rapid decision-making capabilities to ensure quality and pinpoint anomalies. Since vaccines pass through various stages before reaching the vaccination center, a robust cold chain logistics system is essential, requiring continuous monitoring and automated controls throughout the operation. Setting up such an intelligent and integrated system is complex and presents numerous challenges. This includes determining the type and quantity of devices needed, positioning them on goods, ensuring data accuracy, managing large amounts of data, troubleshooting data transmission interruptions, and ensuring long-term reliable operation of devices.

There are currently a variety of temperature monitoring solutions on the market. These include temperature loggers that are skilled at recording data. However, these loggers must be improved to enable real-time data access and remote data sharing. Radio-frequency identification (RFID) and wireless sensor networks (WSN) are emerging as the dominant technologies for cold chain monitoring [48,49]. These technologies increase cold chain efficiency and reduce labor costs. In particular, active RFID tags provide a cost-effective method for monitoring cold chain integrity. They are more efficient, cost-effective, and user-friendly, enabling wireless data transmission for convenient installation and use. These tags record temperature and precise location and synchronize both sets of data to a central database when scanned. Nevertheless, the financial impact of using RFID and WSN remains significant. Consequently, the number of these sensor units tends to be limited. To ensure cost-effectiveness, it is important to allocate them strategically according to the application's specific requirements.

In order to realize the ambient temperature recording of the vaccination process, appropriate hardware and software products need to be developed to provide technical support for the system service. Table 2 summarizes these units and their main functionalities. Hardware devices include four categories of IoT devices.

• Low-power temperature acquisition tags (LTAT),
• Intelligent gateway devices (IGD),
• Cold-chain vehicle equipment (CCVE), and
• Cold-chain incubator equipment (CCIE).

**Table 2:** Specifications of the equipment and the software.

Unit/part name	Functionality	Technology	Location	Additional Features	Sustainable scalability or not
LTAT	Temperature sensing and acquisition, data transmission	2.4 CHz/Low-power MCU	Refrigerators, freezers, and cold storage for vaccines	Long battery life, Wireless communication, Low cost, Small size and easy installation	Yes
IGD	Acquiring the data sent by the LTAT via wireless, data analysis, display, generating alarm information and data transfer functions.	2.4 CHz/4G/embedded technology	Centralized storage area of vaccines	Real-time data processing, Secure data transmission	Yes
CCVE	Collecting the temperature of the vehicle container where the vaccine is stored, obtaining the location information of the vehicle, data display, alarm indication, and uploading the temperature data to the server	4G/BEIDOU/Low power MCU	Cold chain transport vehicle	Temperature control, Route optimization, Low cost, Low power	Yes
CCIE	Collecting the temperature of the incubator where the vaccine is stored, completing the data display, alarm if excessive temperature, obtaining the location information of the incubator, and finally uploading the data to the server	4G/BEIDOU/Low power MCU	Cold chain incubator	Automated alerts, Energy efficiency, Low cost	Yes
Mobile phone apps	Visualization of all information about the vaccine to the vaccinators, including the whole process temperature of the vaccine, and administrators can manage tasks.	Qt technology [50]	Mobile phones of vaccinators and administrators	Friendly interface, Multi-function, High security, Privacy protection, Data analysis and viewing	Yes
Cloud service platforms	Data receiving, parsing and processing, and storage	Web technology, socket communication, network security technology.	Management center server	Supports access to a large number of devices, Efficiency, High reliability, Friendly interface,	Yes
Cloud database	Management of all vaccine information, including all temperature information of the vaccine life cycle	Database technology, security technology and so on	Management center server	High availability, High security, Support for big data processing, etc.	Yes

The following software parts are required for data transmission and management.

• Mobile phone apps,
• Cloud service platforms, and
• Cloud databases.

### 5.1 Hardware design and workflow of the LTAT

The LTAT serves as the system's data source and records the temperature of the environment in which the vaccine is stored. Because vaccine storage spaces are limited and require high levels of sealing, the LTAT must be compact, energy-efficient, and easy to install. The low-power microprocessor CH583 M is, therefore, used as the heart of the LTAT [51]. It has a small footprint, economical power consumption, and a wireless transmission circuit, enabling seamless wireless data transmission over the non-standard 2.4 GHz band. Temperature sensors are available in various forms, including digital and analog variants. While digital sensors exhibit precision, optimized peripheral circuitry, power efficiency and ease of use, analog sensors tend to be more complex and less accurate. The accuracy of LTAT must be superior and less than 0.3 °C. Thus, The TMP112 [52] is selected as the temperature sensor for this research. This sensor features state-of-the-art accuracy, low power consumption, and a minimum operating voltage of 2.0V. Typically, a 1200mAh disposable lithium battery can keep the system running for over three years.

**Fig. 3:**
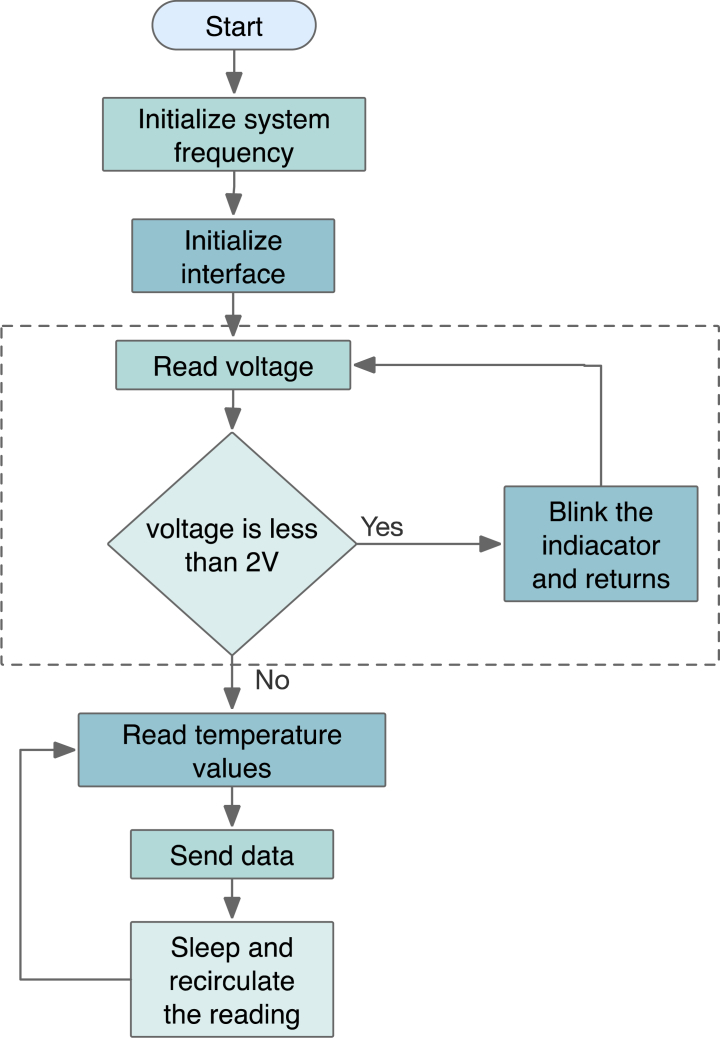
Workflow of the proposed LTAT.

Fig. 3 illustrates the workflow of the LTAT. Once activated, the data sampling frequency is set. Because the temperature within the vaccine storage environment is relatively stable and does not fluctuate rapidly, the LTAT is generally configured to collect data at 1-min intervals to conserve power. This setup ensures that the LTAT remains predominantly in rest or sleep mode. Since the minimum operating voltage of the components in the LTAT is set to 2.0V, it is essential to monitor the battery voltage during each data transfer. If the voltage falls below 2.0 V, an alarm indicator lights up and indicates that the battery needs to be replaced.

It is important to note that the LTAT must be incorporated into any vaccine storage device and is the key hardware component for the comprehensive implementation of the system. Therefore, cost management is of utmost importance in real system applications. The associated costs of the hardware components required for the LTAT are summarized in Table 3. As indicated in Table 3, the estimated cost of each LTAT is approximately $3.

**Table 3:** Approximate cost of LTAT.

Item (Model)	Quantity	Unit Price (USD)
Microcontroller (CH583 M)	1	1
Temperature sensor (TMP112)	1	0.5
LED	1	0.01
PCB board	1	0.5
Resistors and capacitors	20	0.1
Lithium battery (1200 mAh)	1	1

### 5.2 Hardware design and overall functionality of IGD

The IGD serves as a data management and transmission unit and acts as a communication channel between the LTAT and the cloud servers. Using the 2.4 GHz frequency band, the IGD can receive temperature readings from multiple LTATs simultaneously. It handles data analysis, storage and real-time display, providing on-site managers with instant access to critical temperature metrics. In addition, the IGD repackages new data and transfers it to cloud servers for central management. To perform these functions and maintain consistent system performance, the IGD was designed around the T113-S3 core [53]. This system features a 7-inch capacitive touchscreen that allows real-time data visualization, access to historical records, alarm logs, and graphical data representations. The IGD is also equipped with the 4G Cat.1 EC200U module [54], ensuring a stable cloud server connection. This module facilitates the transfer of all temperature data collected by the LTATs to the cloud server, thereby enabling remote access to the data. To ensure the long-term stable operation of the device, the IGD contains an integrated 4800 mA lithium battery. This feature ensures that the device can maintain its standard operation for over 24 h, even in the event of an unforeseen power interruption.

**Fig. 4:**
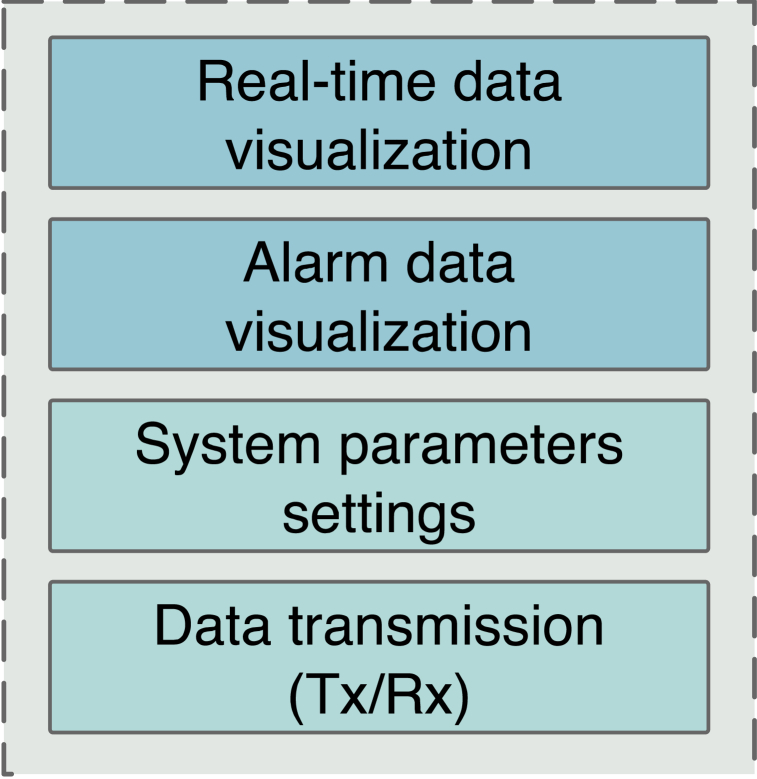
Software functional structure of the IGD.

The device software uses the Linux 5.4 platform, QT technology to create a user-friendly human-machine interaction interface, and MySQL database technology to achieve local data recording and management. The functional structure of the software is shown in Fig. 4.

### 5.3 Hardware design scheme and workflow of the CCVE

**Fig. 5:**
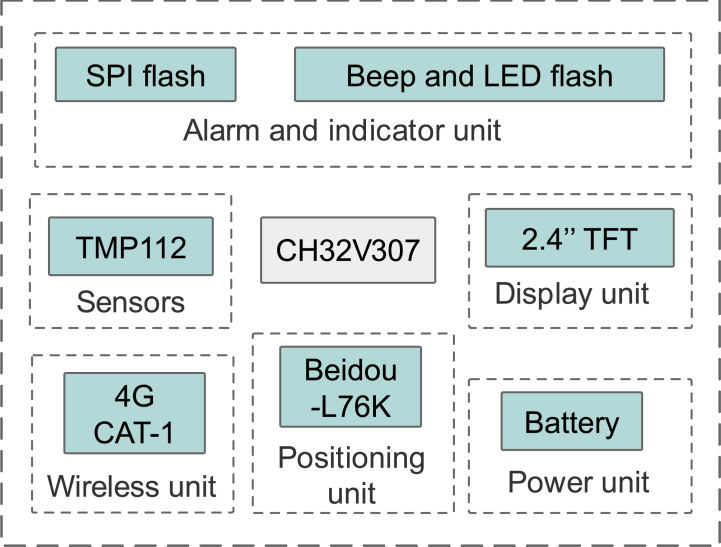
Hardware diagram of the CCVE.

Transport plays a central role in the vaccine life cycle. It is important to maintain the correct ambient temperature during transport. In this context, the CCVE is primarily integrated into transport vehicles for the cold chain of vaccines. Its main function is to enable real-time temperature monitoring and transmission throughout the transportation of the vaccine while providing instant location updates. The CCVE has two temperature monitoring units connected via cable channels and positioned in the transport container. T. The controller is strategically placed in the cab to ensure convenience and instant access to important information so the driver can easily monitor temperature levels. CH32V307 [55], a low-power microcontroller unit (MCU), is one of the main units in CCVE. This device features a 2.4-inch TFT LCD that displays temperature data and geographic location information. T-he device integrates the 4G Cat-1 module via the TTL interface to ensure that the data is consistently uploaded to the cloud servers. In addition, the Beidou L76K positioning module enables real-time vehicle location tracking. The architecture diagram of this system is shown in Fig. 5.

**Fig. 6:**
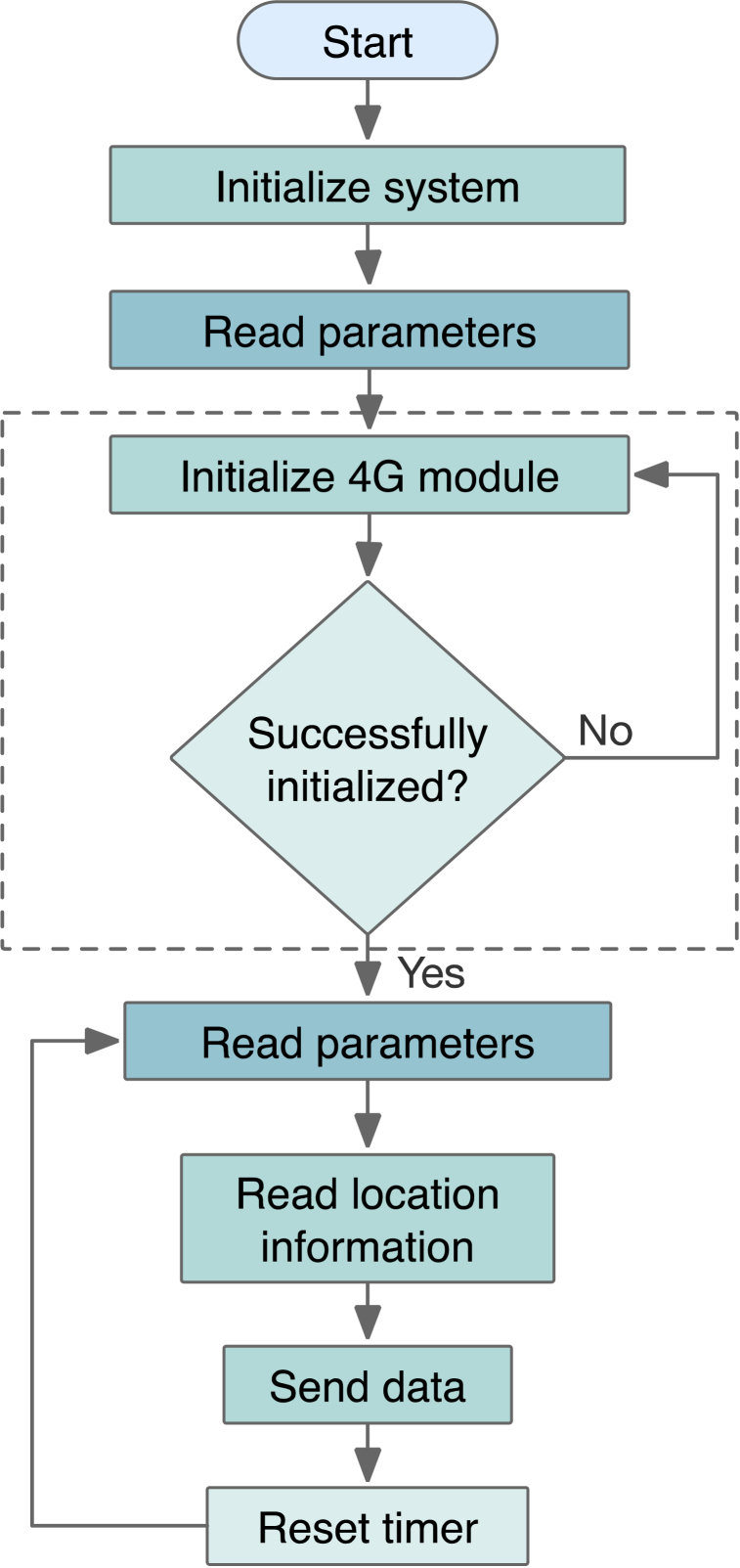
Workflow diagram of the CCVE.

Fig. 6 shows the overall system workflow diagram. Once the device is activated and the system is initialized, it retrieves its parameter settings. The 4G CAT1 module then begins setting up. After successful initialization, the system establishes a connection to the cloud server. It is crucial to synchronize the system time with the server before proceeding with temperature data collection. In addition, the system collects vehicle location details every minute to optimize power consumption and communication effort. At the same time, our proposed system uploads the temperature and geographical data to the server at regular intervals.

### 5.4 Hardware design scheme and workflow of the CCIE

The CCIE is primarily designed for short-distance transport of limited quantities of vaccine. In certain situations, there is an urgent need to distribute a minimum amount of vaccine. This device efficiently meets these requirements by ensuring rapid vaccine transport while consistently maintaining the vaccine within a specified and safe temperature threshold. The equipment typically includes an isolation box and a collection controller. The insulated box should be lightweight and have excellent sealing and insulating properties.

On the other hand, the collection controller generally includes an external temperature sensor, an MCU, a display unit, and a 4G module. This setup makes it easier to collect data, which is then transferred to the cloud server. The hardware and software specifications for the CCIE should be consistent with those of the CCVE.

## 6 IoT-enabled framework

To minimize the loss of vaccines during transportation and storage, an IoT framework based on the above equipment was proposed to monitor the ambient temperature parameters of real-time vaccines automatically. In addition, this study aims to demonstrate that wireless network systems based on IoT technology can function adequately with sufficient flexibility, signal loss protection, and energy autonomy. The system should be designed to measure variables in real-time and reduce power consumption to ensure maximum battery autonomy. The system was designed and modularized to improve its stability, maintainability, and sustainability.

**Fig. 7:**
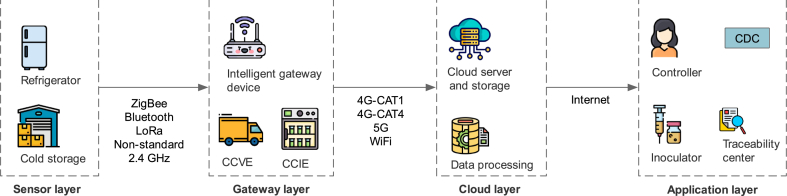
Proposed IoT-enabled framework.

As shown in Fig. 7, most modern IoT systems consist of the following four layers: These layers cover the entire vaccine data lifecycle, from collecting temperature data to analyzing, storing, and visualizing the vaccine recipient's information. Additionally, the cloud allows vaccinators to view complete details of their vaccines throughout the vaccine lifecycle, such as the temperature of the storage environment.

• *Sensor/perception layer:* This layer mainly consists of energy-saving temperature detection sensors that are installed in cold storage, refrigerators, or freezers for storing vaccines. Besides, wireless protocols such as Bluetooth, Wi-Fi and ZigBee transmit recordings to subsequent layers.
• *Gateway layer:* Due to IoT devices' storage and computing limitations, raw data is sent to the next layer. You can use mobile modules or dedicated access points (APs), which are usually more powerful than sensor nodes. They can perform basic pre-processing tasks, such as quickly reviewing and storing data and conducting preliminary investigations based on artificial intelligence. Furthermore, these middleware devices use the Internet to transmit data collected by the sensors to the cloud layer. In addition, IGD, CCVE, and CCIE belong to this layer.
• *Cloud layer:* This layer plays a vital role in this framework, firstly, it completes the reception of data and remote access control of various terminal devices, realizes real-time data uploading of terminal devices, such as IGD, CCVE, CCIE, to ensure the integrity and security of data. Secondly, the data received can be processed and analysed in real-time. received data can be processed and analysed in real time, which helps to extract valuable information and provide support for decision-making.
• *Application layer:* Managers, manufacturers, vaccinators, and other stakeholders can access the temperature data and additional vaccine information. With the vaccination information, managers can better monitor the occurrence and prevention of epidemics. Examples include the allocation and status of vaccines.

The framework has a temperature-sensing layer that is dedicated to collecting temperature readings from various vaccine storage environments. This data is then forwarded to a gateway device for consistent monitoring. In the event of temperature irregularities, alarm signals can be sent to vaccine managers for immediate intervention to reduce vaccine waste or spoilage. All of these measurements are archived in a cloud database. Given limited device resources and to avoid delays during unmonitored night hours, SMS notifications are managed at the cloud level rather than on the client side. This makes it easier to notify multiple vaccine managers at the same time. The resulting temperature analysis enables medical supervisors to take decisive action. This arrangement ensures a system that is not dependent on an administrator's processing capacity and avoids potential delays.

## 7 Proof of concept for IoT-enabled framework

In order to further validate the feasibility and scientificity of the framework and ensure that the constructed framework can achieve the expected results in practical applications, the validation objectives are firstly determined to clarify the functional and non-functional requirements that the framework needs to satisfy, which include validating the LTAT's acquisition temperature accuracy, power consumption and stability of low-temperature operation, verification of IGD data reception and transmission stability, power failure alarm function, verification of CCVE and CCVE data acquisition accuracy and stability, positioning function. and verifying the functionality, operability and stability of the mobile phone APP. It also further verifies the performance, stability and alarm and warning function of the cloud platform system. Based on the above validation objectives, a specific validation programme is designed, including test cases, test environment, test data, etc. Ensure that the test scenarios cover all aspects of the framework, including main functions, boundary conditions, exception handling, etc. Determine the specific test environment and select a community vaccination centre as the actual application test scenario, which contains five vaccine storage freezers, two cold chain insulation boxes, one cold chain transport vehicle from the city CDC, and two vaccine managers at the centre.According to the analysis of the actual environment, it is determined to install a LTAT in each vaccine storage freezer, install a set of IGD in the place where the freezer is located, install a set of CCIE in cold chain insulation box, and install a set of CCVE on the cold chain transport vehicle, and each equipment has been calibrated in the professional metrology company for accuracy before installation and meets the system accuracy requirements: temperature <0.3 °C, humidity <5%. After the installation of hardware equipment is completed, account information is established in the platform system for two managers, and the mobile phone information of the managers is recorded to receive abnormal alarm information. After the system installation and deployment is completed, it starts to execute the test according to the test programme, the freezer equipment works 7*24 h uninterruptedly, the interval of uploading data for each equipment is set at 1 min, and the test time cycle is 7 days, the insulation box equipment runs for a total of 6 h for 3 times, and the cold-chain transport vehicle runs for 4 h. At the end of the test, the data packet loss rate was analysed, and the packet loss rate of each measurement point was less than 1%, which meets the system's requirement of less than 2%, and verifies that the 2.4GHZ and 4G CAT-1 wireless transmission adopted by the system works stably, and provides a guarantee for the stable transmission of data. During the test, by simulating the power supply disconnection of the gateway equipment and the abnormal temperature of the freezer, the platform system generates alarm information and sends the alarm information to the two managers in a timely manner; during the test period, the two staff members can use their accounts at any time through the mobile phone app or the platform server to remotely view all the real-time data of the measurement points, historical data, alarm data and so on. Through the above illustration of the implementation process and final results, the framework in real-time monitoring performance, stability, ease of use, scalability and other aspects of the preset indicators to meet the requirements. It can ensure that the framework can achieve the expected results in practical applications and provide a strong guarantee for the successful implementation of the project.

## 8 Application cases and analysis

A given province with a large population has a significant annual consumption of various vaccines. Traditional approaches to vaccine management have been used in the past, resulting in fragmented information and reduced efficiency. Poor monitoring and management of vaccines in circulation often led to mishaps. To remedy this situation, an IoT-based vaccine monitoring system is designed to be centrally coordinated by the provincial disease control agency. This system primarily focuses on the vaccine's storage temperature and monitors the vaccine's distribution and administration by integrating and managing the data throughout the vaccine circulation process in the province. This is achieved through the use of established pharmaceutical oversight and management operating models.

As a result, a comprehensive vaccine traceability information chain has been established within the province, providing regulatory authorities with easy access for queries and retrieval. Any anomalies can be detected by compiling and reviewing critical data from the vaccine circulation chain, providing important information support for regulatory actions. The following sections cover the user requirements design of the system, the design of the business functionality, and an analysis of the device requirements.

### 8.1 System user requirements design

The users of this vaccine regulatory platform are classified into two categories: platform operation and maintenance users and platform users.

(1) *Platform operation and maintenance.* Platform operation and maintenance users are mainly regulatory personnel from the provincial drug regulatory bureau, provincial/municipal disease control and prevention centers, staff from various vaccination units, and vaccination personnel. These users are mainly responsible for early equipment demand planning and installation and managing equipment information input. The structural design, daily operation, maintenance, and management of the platform are responsible for establishing organizational structures, creating personnel roles, managing various types of data, and managing user authorization to ensure regular and stable operation of the platform.
(2) *Platform users.* These users have four main roles: regulatory personnel from the provincial drug regulatory bureau, regulatory personnel from the provincial/municipal centers for disease control and prevention, staff from vaccination units, and vaccination personnel. Users have different levels of authority based on their job responsibilities, and other roles can use the corresponding functional modules within their jurisdiction.

Personnel at the provincial and municipal disease control and prevention centers oversee the intake, dispatch, and inventory management of vaccines stored in cold storage within their affiliated units. They manage the logistics of vaccines, including their admission and dispatch from these units, and record details such as inventory on the platform. Officials at the provincial level can access and retrieve this information. These supervisors can monitor temperature data during the storage and transit of all vaccines throughout the province. On the other hand, staff at individual vaccination units handle the storage and utilization of vaccines within their domain. They are tasked with updating the platform with data related to their unit's storage, inventory, and vaccination records. Additionally, they can access temperature data for all the vaccines specific to their team.

Supervisors can view the analytical outcomes of vaccine data within the province via the platform. This gives them a clear understanding of vaccines' current distribution and consumption patterns across the region. Furthermore, they can institute a temperature data alert system by setting specific warning criteria. In a vaccine-related emergency, regulatory officials can swiftly access the distribution data of the affected vaccine, pinpoint its location, and liaise with the relevant party for offline intervention, facilitating efficient vaccine recalls and reallocations.

### 8.2 System business function design

The regulation of vaccines encompasses the entire lifecycle of the vaccine, with the temperature of the storage environment being of paramount importance during its distribution. This vaccine oversight platform was developed in compliance with the guidelines outlined in the Vaccine Management Law and in alignment with the oversight requirements of officials from provincial pharmaceutical regulatory bodies. An analysis of the system's business operations is provided below.

(1) The platform gathers and evaluates real-time data concerning the cold chain equipment's temperature, humidity, and other environmental conditions throughout the vaccine distribution process. In the event of anomalies, it swiftly notifies regulatory officials to implement emergency interventions, ensuring the province's vaccines remain high quality and safe during storage and transit.
(2) The platform logs and oversees data related to the entry, exit, stock levels, distribution, and utilization of vaccines across all institutional levels within the province. Additionally, it tracks the flow of vaccines throughout the region to guarantee that the location and journey of each vaccine batch remain traceable.
(3) The platform calculates and investigates critical data, including vaccine stock levels and consumption within the province. Using predefined alert mechanisms, it flags potential risks, such as dwindling vaccine inventory. These aid regulatory officials in making informed decisions, such as reallocating resources, to ensure consistent vaccine availability.
(4) The platform collaborates with vaccine manufacturing entities to access fundamental traceability details, including batch numbers and producer information. This ensures the province has a clear trace of each vaccine's origin.

### 8.3 System equipment requirements analysis

**Fig. 8:**
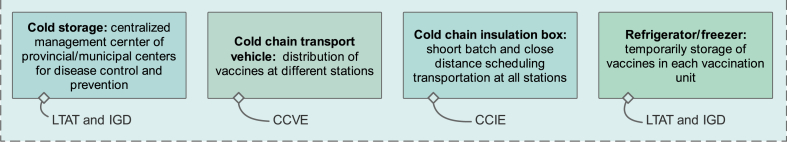
Storage location and equipment requirements.

Vaccines, throughout their circulation process, are primarily stored in centralized management facilities and cold storage warehouses at provincial and municipal disease control and prevention centers. Moreover, they are transported in refrigerated trucks at various distribution levels, stored in insulated boxes for emergency small-batch vaccine allocations, and placed in multiple temperature-specific refrigerators or freezers at diverse vaccination centers at all stations. To ensure real-time temperature monitoring at each of these storage points, distinct IoT-enabled devices are essential. Fig. 8 depicts the storage locations and their corresponding equipment needs.

Vaccines are distributed across various locations and have multiple storage points. To reduce cost, enhance system maintainability, and ensure the best equipment setup for each vaccine storage site, we determine the necessary number of devices for each storage setting as follows:

***Cold Storage***. Cold storage is generally a central location purchased by provincial/municipal disease control and prevention centers from vaccine manufacturers. The cold storage space varies, and the required monitoring temperature points are different. Therefore, the necessary labels for temperature are various. The number of temperature labels needed for cold storage is shown in the following Expression 1:

NLTAT=U{H,S,ε},

where H denotes the height of the cold storage, S represents the cold storage area, and ε is the cold storage interference source coefficient.

Due to the relatively low temperature of the cold storage, the storage environment for some particular vaccines is around −40 °C. It is generally installed outside the cold storage to ensure the regular operation of the gateway. The gateway receives temperature data from multiple LTATS in areas with concentrated cold storage. The number of gateways is represented by the following Expression 2.

NIGD=U{NLTAT,D,ε},

where and NLTAT denotes the number of LTAT that are less than 10, D represents the distance between LTAT and IGD; ε denotes the interference source coefficient of the environment.

(1) *Cold-Chain Transport Vehicles.* This unit is typically designed to transport large quantities of vaccines. Transport vehicles usually have standard devices with two wired temperature sensors. Additional temperature sensors should be installed for specific scenarios where the box's volume is very large. A maximum of four temperature sensors can be connected to each on-board device.
(2) *Cold-chain Insulation Box Equipment.* This equipment is commonly used for emergency transfers and allocating small quantities of vaccine when demand is low. The community centers for CDC and prevention generally administer and make it available for uniform use.
(3) *Refrigerator/Freezer.* Due to the diversity of vaccines and their specific temperature storage requirements, vaccination stations typically use refrigerators or freezers. This results in the highest number of storage options for vaccines. While these storage devices are typically distributed and managed centrally in a single room, they can also be distributed as needed. According to vaccine storage guidelines, certain storage rooms must have temperature monitoring. Therefore, the LTAT should be placed in a freezer in different temperature zones. Consequently, in this setting, the number of LTATs correlates with the number of independent storage sections at different temperatures. The number required for IGDs is similar to that in cold storage scenarios. The formula for determining the number of IGDs remains consistent with Expression 2.

The provincial drug administration counted all vaccine storage locations in the province. The quantity of equipment required according to the above rules is summarized in Table 4.

**Table 4:** Quantity of various equipment.

Device Name	Number of devices
LTAT	24172
IGD	5672
CCVE	1465
CCIE	346

The software platform used by the IoT Vaccine Monitoring System is implemented on servers monitored by the Operation and Maintenance Department of the Provincial Drug Regulatory Authority. These IoT devices will be uniformly distributed to administrative departments at each vaccine storage location, where they will be installed and fine-tuned as needed. We find that leveraging IoT technology for the integrated design of the system has significantly improved its performance, outperforming traditional approaches to vaccine management. Table 5 summarizes the above two approaches in various aspects, such as power consumption, accuracy, real-time responsiveness, alarm systems, installation processes, environmental aspects, and sustainable development.

**Table 5:** Performance comparison.

Performance	IoT system	Traditional systems
Consumption	Low	High
Accuracy	High	Low
Real-time	Yes	No
Real-time Alarm	Yes	No
Installation method	Simple	Complex
Environmental protection	High	Low
Development sustainability	Yes	No

### 8.4 System disadvantages

The main objective of the vaccine cold chain management framework is to ensure that vaccines remain within a certain temperature range throughout the entire process, from production to consumption, to guarantee product quality and safety. However, despite its many advantages, there are some possible disadvantages. The following are some of the possible disadvantages:

Technical complexity: Cold chain management systems usually involve multiple technologies and equipment, such as temperature monitoring equipment, data analysis software, and cold chain transport vehicles. The complexity and diversity of these technologies and equipment may lead to difficulties in operation and maintenance.

Cost issues: The construction and operation of a vaccine cold chain management system requires significant investment, including the purchase of equipment, software licences, and staff training. This may become a challenge for some regions or organisations with limited resources.

Personnel training and management: The operation and maintenance of a vaccine cold chain management system requires specialized knowledge and skills. However, personnel training may be inadequate or poorly managed, leading to operational errors or negligence, which can affect the quality and safety of vaccines.

## 9 Conclusions

Optimizing the management of the vaccine cold chain is a pressing issue as the proper functioning of the vaccine cold chain ensures vaccine safety, prevents the onset of disease and reduces or stops the spread of outbreaks. This study designed a new framework based on Internet of Things (IoT) technology to improve vaccine cold chain management. Since the COVID-19 outbreak, many new crown vaccine cold chain accidents have occurred, leading to the scrapping of a large number of relatively scarce new crown vaccines and exposing many problems in vaccine cold chain management. By analyzing the causes of several cold chain incidents involving COVID-19 vaccines, a low-power and low-cost temperature and humidity collection device, a multifunctional intelligent gateway device, a mobile phone APP, and a cloud platform management platform, a vaccine cold chain management framework.

Furthermore, this article thoroughly studies hardware design and analyzes the features and costs associated with multiple devices. Additionally, we provide detailed design plans for IoT devices, covering aspects such as chip selection, circuit design, and software programming. To ensure high availability, these devices guarantee real-time data transfer, security, scalability, adaptability, and minimal latency within the system architecture. In fact, the proposed IoT-based architecture significantly reduces vaccine distribution risks, increases efficiency, and guarantees security.

Finally, this paper describes the deployment of the overall framework in a provincial disease control center and proposes the system software's functional requirements and specific design. Moreover, the research assesses vaccine storage locations and quantifies the need for various IoT devices. We also compare our proposed solution with traditional vaccine management systems. In summary, the IoT framework presented in this study improves the precision and security of vaccine management and leads to its practicality, environmental friendliness, and sustainability.

Through practical application deployment, the evaluation results show that by implementing the vaccine cold chain management framework described in this paper, the collection of vaccine-related information, the cost of vaccine management, and the monitoring of the vaccine storage environment have been improved, and the number of vaccine accidents has been reduced, while the pollution of the environment has been reduced. After several years of long-term stable use, the following three recommendations are made on how to improve the stability and effectiveness of the framework: 1. Ensure the stability and sustainability of the vaccine storage temperature and humidity monitoring equipment, and reduce the cost and power consumption of the equipment, the monitoring equipment is the source of data and the basis of the management framework. 2. Ensure the scalability and sustainable updating of the framework-related software, the software is an important means of improving the management framework, and as the technological progress and increased demand, the corresponding software requirements function will also be continuously updated.3, the management framework implementation of the management personnel responsibility allocation, to ensure that the vaccine cold chain accident can be guaranteed even if the accurate treatment.

Nevertheless, this study has some limitations. This study mainly focuses on the pre-development and deployment of the framework to improve the level of real-time monitoring of vaccine storage temperature and humidity in vaccine cold chain management, and effective temperature warning and alarming. However, there is no effective use of the data produced by the framework, and in future research, blockchain and machine learning is introduced to analyse and process the data to better predict the risk of vaccine storage, and to optimize vaccine cold chain management, vaccine cold chain transport, and vaccine cold chain distribution.

## Data availability

Data used in this study are available from the corresponding author on reasonable request.

## CRediT authorship contribution statement

**Shaojun Jiang:** Writing – review & editing, Writing – original draft, Software, Project administration, Conceptualization. **Sumei Jia:** Project administration. **Hongjun Guo:** Writing – review & editing.

## Declaration of competing interest

The authors declare that they have no known competing financial interests or personal relationships that could have appeared to influence the work reported in this paper.
